# Integration of Artificial Intelligence into the Approach for Diagnosis and Monitoring of Dry Eye Disease

**DOI:** 10.3390/diagnostics12123167

**Published:** 2022-12-14

**Authors:** Hee Kyung Yang, Song A Che, Joon Young Hyon, Sang Beom Han

**Affiliations:** 1 Department of Ophthalmology, Seoul National University College of Medicine, Seoul National University Bundang Hospital, Seongnam 13620, Republic of Korea; 2 Department of Ophthalmology, Kangwon National University School of Medicine, Kangwon National University Hospital, Chuncheon 24289, Republic of Korea

**Keywords:** artificial intelligence, dry eye disease, deep learning, machine learning

## Abstract

Dry eye disease (DED) is one of the most common diseases worldwide that can lead to a significant impairment of quality of life. The diagnosis and treatment of the disease are often challenging because of the lack of correlation between the signs and symptoms, limited reliability of diagnostic tests, and absence of established consensus on the diagnostic criteria. The advancement of machine learning, particularly deep learning technology, has enabled the application of artificial intelligence (AI) in various anterior segment disorders, including DED. Currently, many studies have reported promising results of AI-based algorithms for the accurate diagnosis of DED and precise and reliable assessment of data obtained by imaging devices for DED. Thus, the integration of AI into clinical approaches for DED can enhance diagnostic and therapeutic performance. In this review, in addition to a brief summary of the application of AI in anterior segment diseases, we will provide an overview of studies regarding the application of AI in DED and discuss the recent advances in the integration of AI into the clinical approach for DED.

## 1. Introduction

The advancement of computer science and the availability of big data has enabled the emergence of artificial intelligence (AI; The abbreviations were summarized in Table 1), which has led to a technological revolution significantly affecting many aspects of our daily life [1,2,3,4]. The application of AI in the field of medicine is expanding rapidly [5], mainly due to the advancement of machine learning (ML) that can be utilized for the analysis of medical images and patient data, diagnosis of diseases, and prediction of treatment outcomes [6].

ML is a paradigm of AI that systematically allows computer algorithms to adapt according to a large amount of raw input data and make predictions or determinations using the learned patterns [1,7,8]. The method can be roughly divided into conventional machine learning (CML) and deep learning (DL) [8]. CML algorithms, such as the support vector machine (SVM), random forest (RF), decision tree (DT), and linear regression and logistic regression, generally do not involve large neural networks [8] and have been applied for the construction of predictive algorithms for the diagnosis or classification of diseases based on data from medical records or population-based studies [9]. DL has usually been applied for the analysis of multimedia datasets, including images, sound, and videos [7,8], and involves large neural networks composed of multiple neuron-like layers of algorithms, such as artificial neural networks (ANNs), recurrent neural networks (RNNs), and convolutional neural networks (CNNs) [7,8].

In ophthalmology, AI has initially been applied for the analysis of fundus photographs and optical coherence tomography (OCT) images; thus, previous studies have mostly focused on the integration of AI into the diagnostic approach of posterior segment diseases, such as diabetic retinopathy, glaucoma, macular degeneration, and retinopathy of prematurity [5,10,11,12,13,14]. However, as DL algorithms can be utilized for the analysis of imaging the data of anterior segment structures, such as anterior segment photographs (ASPs), anterior segment OCT (AS-OCT) images, specular microscopy, corneal topography, in vivo confocal microscopy (IVCM), infrared meibography, and tear interferometry [2], AI is also expected to assist in the diagnosis and monitoring of various anterior segment diseases [15]. Recently, many studies have been conducted on the application of AI in various anterior segment diseases [2].

## 2. Application of AI in Anterior Segment Diseases

An ML model using an SVM showed an accuracy of 97% (97% sensitivity and 99% specificity) for the diagnosis of anterior segment disorders, including arcus senilis or cataracts, suggesting the potential of AI as a screening tool [16]. Subsequent studies revealed that AI-based algorithms using ASPs could be useful for the detection and grading of cataracts and even the classification of referable cases [17,18,19,20]. AI-based algorithms were also shown to enable a more accurate prediction of refractive outcomes after cataract surgery, which is crucial for optimal visual outcomes [21,22,23].

AI can also be helpful for the diagnosis and management of various ocular surface diseases. Recent studies have shown that DL algorithms can automatically detect the presence of pterygium, classify its grade, and identify referable cases with high accuracy [24,25,26], suggesting that AI may be used as a simple screening tool for the identification of pterygium cases that need a referral to ophthalmologists in community screenings [26]. Kim et al. [27] introduced an AI model using a bagging tree that may enable the automated quantitative analysis of histopathological images of pterygium. Regarding corneal infection, Saini et al. [28] reported that an AI model using an ANN showed a high accuracy of 90.7% for the classification between bacterial and fungal keratitis, outperforming the prediction rate of 62.8% by the clinical investigator. AI enables the automated segmentation of corneal ulcer areas in ASPs, which may enhance the reliability of the quantitative assessment of the treatment response in patients with corneal ulcers [29,30,31]. AI can also be helpful for the identification of hyphae in IVCM images, indicating its potential as a powerful tool for a non-invasive and accurate diagnosis of fungal keratitis [32,33,34]. Another Al model based on a K-means clustering model showed high accuracy (100%) for the automated detection of iris tumors [35].

The integration of AI into evaluation algorithms based on corneal topography has been proven to improve the accuracy of the discrimination of keratoconus, even in early cases [36,37,38,39,40]. Indices generated using the RF model based on data from Pentacam HR (Oculus, Wetzlar, Germany) showed significantly superior accuracy for the detection of keratoconus compared to other non-AI methods [36,37,38]. A DL model based on a CNN using data from a corneal topographic map also showed a high accuracy of 96.4%, with 94.1% sensitivity and 97.6% specificity [41]. Kamiya et al. [42] reported that a DL model based on color-coded maps obtained using AS-OCT data showed an accuracy of >97% for the differentiation of keratoconus from normal corneas. For the treatment of keratoconus, a DL model using an ANN was suggested to be helpful for predicting the quality of vision after intracorneal ring implantation [43]. AI models can improve the prediction accuracy of the screening of patients at high risk of developing iatrogenic ectasia after corneal refractive surgery, including laser-assisted epithelial keratomileusis (LASEK), laser in situ keratomileusis (LASIK), and small incision lenticular extraction (SMILE) [37,44,45]. Lopes et al. [37] revealed that an index developed using the RF model showed superior accuracy for the classification of these patients to conventional methods. Cui et al. [46] reported that an ML model using a multilayer perceptron algorithm improved the efficacy of the nomogram prediction in SMILE, suggesting that AI can be helpful for optimizing visual outcomes after refractive surgery [46].

AI may be useful for the automated evaluation of the corneal endothelium, which might be useful for the long-term assessment of corneal grafts [47,48,49,50,51]. DL algorithms based on U-net enabled the rapid and accurate segmentation of corneal endothelial cells [47,48,51,52]. Treder et al. [53] reported that a DL model using a CNN could allow for the automated detection of Descemet membrane endothelial keratoplasty (DMEK) graft dislocation based on AS-OCT images, with a high accuracy of 96%. Hayashi et al. [54] introduced a DL model that enabled the automated clinical judgment of the need for rebubbling in cases with detached grafts after DMEK with high accuracy (the area under the receiver operating characteristic curve [AUC], 0.96; sensitivity, 96.7%; and specificity, 91.5%), suggesting that the application of AI may improve the survival rate of corneal grafts.

## 3. Application of AI in Diagnosis and Treatment of Ded

Dry eye disease (DED) is one of the most common diseases, with a prevalence of 10–40% worldwide [55,56,57]. It is a multifactorial ocular surface disease characterized by the loss of tear film homeostasis, such as hyperosmolarity and instability of the tear film [58]. Symptoms of DED, such as ocular discomfort, redness, pain and grittiness, foreign body sensation, and visual blurring, may interfere with daily activities, including reading, using digital devices, and driving [58,59,60]. Thus, the disease can result in significant impairment in quality of life [61].

The lack of correlation between dry eye symptoms and signs has often been reported, which renders the diagnosis and monitoring of DED challenging [62,63,64]. The heterogeneous nature of the pathophysiology of DED and the individual difference in the perception of dry eye symptoms may partly result in this discrepancy [65]. In addition, the limited reliability of the currently available diagnostic tests for DED may also play a role [65]. Traditional dry eye tests, including the Schirmer test and fluorescein tear film break-up time (BUT), often show poor reliability and reproducibility [66]. The absence of an established consensus on the diagnostic criteria for DED also makes its diagnosis difficult [67]. New imaging modalities, such as infrared meibography, tear interferometry, IVCM, AS-OCT, and non-invasive tear BUT meibography, can allow for the visualization and assessment of the ocular surface and tear film. However, although these modalities are objective, their interpretation may often depend on the subjective judgment of the examiners.

To overcome these challenges, AI can be applied to the analysis of DED tests and the construction of protocols for the diagnosis and monitoring of the disease. Many studies have reported promising results of AI-based algorithms for the diagnosis of DED and analysis of the results of DED tests. Herein, we aim to provide a comprehensive overview of the studies and discuss the recent advances in the integration of AI into the approach for the diagnosis and treatment of DED.

### 3.1. Application of AI for Analysis of Medical Data

In 1999, Grus et al. [68] proposed a method for an ML-based analysis of the electrophoretic patterns of tear proteins using an ANN and showed that an AI-based analysis of the tears was effective for the detection of DED. In 2020, Nam et al. [69] developed an explanatory model of DED using an ML-based model and network-based factor analysis of the data from a large population study, the Korea National Health and Nutrition Examination Survey (KNHANES). In this study, continuous factors were classified using DTs, and important factors were selected using the least absolute shrinkage and selection operator (LASSO) regression [69]. These factors were then used for the training of a survey-weighted multiple logistic regression, and the interrelationship of the DED-associated factors was evaluated using the network graphs of the correlations between these factors [69]. This model revealed that important risk factors of DED included female sex, corneal refractive surgery, cataract surgery, depression, psychological stress, age of 54–66 years, rhinitis, lipid-lowering medication, and omega-3 intake, in which age (54 to 66 years) had the highest centrality in the network of the correlations between these risk factors [69]. Dros et al. [70] proposed an ML-based algorithm for the identification of primary Sjögren’s syndrome using routine healthcare data. In this study, the ML model was developed using logistic regression (LR) and an RF for the classification of patients with and without primary Sjögren’s syndrome, which attained an AUC of 0.82 (LR) and 0.84 (RF), a sensitivity of 72.3% (LR) and 70.1% (RF), and specificity of 74.0% (LR) and 77.9% (RF) [70].

### 3.2. Analysis of ASPs and Videos Using AI

Photographs or videos of anterior segment structures, such as the cornea, conjunctiva, and eyelid, taken during a slit lamp examination can provide information regarding the diagnosis of DED and monitoring of its treatment. However, assessment of the photos and videos is often less reproducible and repeatable because of the lack of tools for reliable, objective, and quantitative analysis, in which the application of AI is expected to be helpful for the development of reliable interpretation tools.

In 2007, Yedidya et al. [71] developed a method for the automated detection of dry regions in videos recorded during a fluorescein tear film BUT test using the RANdom SAmple Consensus (RANSAC) algorithm. In a subsequent study, they introduced a protocol in which dry areas were segmented using a multi-label graph-cut algorithm on the 3D spatio-temporal data converted from a video sequence recorded during the test [72]. The growth of the dry areas was measured using material recovery facilities (MRFs) and expressed as a time-evolving map of the degrees of dryness [72]. These methods enabled the accurate detection and quantitative measurement of the dry areas, which can be helpful for the evaluation of the severity of DED [71,72]. Recently, Zheng et al. [73] developed a DL-based algorithm for blink analysis using videos recorded by a Keratograph^®^ 5M (Oculus Optikgeräte GmbH, Wetzlar, Germany). In this study, the frequency of the incomplete blinking measured using the AI model was closely associated with the signs and symptoms of DED, suggesting the potential of the DL algorithm as a diagnostic tool for DED [73].

In 2022, Wang et al. [74] proposed a DL model constructed and based on VGGNet-13 for the automated identification of lid margin abnormalities, which are potentially associated with DED, using ASPs. This DL model achieved excellent accuracy for lid margin abnormalities with high sensitivity and specificity, which were as follows: lid margin irregularity (AUC, 0.977; sensitivity, 0.930; and specificity, 0.938), vascularization (AUC, 0.980; sensitivity, 0.923; and specificity, 0.961), hyperkeratinization (AUC, 0.964; sensitivity, 0.948; and specificity, 0.948), and meibomian gland (MG) plugging (AUC, 0.968; sensitivity, 0.979; and specificity, 0.867) [74]. These findings suggest that the integration of AI into the evaluation protocol of ASPs may be helpful for the diagnosis and treatment of DED [74].

Chun et al. [75] developed a digital image analysis technique that could be useful for the objective assessment of corneal staining. In this software, they applied a combination of the difference of Gaussian’s edge detection for morphologic features, the red–green–blue systems, and the hue–saturation–value color model for color detection and used Otsu thresholding, a median filter, and a contrast-limited adaptive histogram equalization for enhancing the contrast [75]. Pellegrini et al. [76] showed that their objective technique of digital image analysis based on ASPs was useful for the objective quantification and morphological characterization of corneal staining in DED and for differential diagnosis of Sjögren syndrome and ocular graft-versus-host disease. Park et al. [77] introduced an automated image analysis algorithm for the objective assessment of a conjunctival injection that showed an excellent correlation with subjective grading by ophthalmologists. Subsequent studies have proven that the image analysis technique can be a useful tool for objective and precise quantification of conjunctival injection [78,79,80], which is conceivably helpful for monitoring DED. Kim et al. [81] developed software for the automated assessment of corneal neovascularization and showed that it was more reproducible and time-saving than the manual method. However, AI has not been used in these studies. We believe that the integration of AI into these automated image analysis techniques based on ASPs is expected to be helpful for the precise and accurate assessment of the disease activity of DED and monitoring of the treatment response. However, it is not uncommon to find patients without any visible changes in the cornea and conjunctiva, despite significant dry eye symptoms [62,63,64]. Thus, clinicians should not solely depend on the analysis of AI for the diagnosis and treatment of DED.

We have recently shown that AI-based algorithms for the analysis of histopathological images might be a reliable method for the quantitative assessment of the histopathological features of pterygium [27], suggesting that AI can also be helpful for the analysis of histopathological samples, such as impression cytology specimens.

### 3.3. Analysis of Meibography Images Using AI

Infrared meibography enables the visualization of the two-dimensional silhouette of MGs, and it can provide information regarding the amount of the MG’s dropout, the area of the MG’s acini, and the length of the MG’s duct [82,83]. Hence, the device may allow the evaluation of the severity of meibomian gland dysfunction (MGD) and DED associated with MGD [83]. For a more precise assessment of the meibography images, semiautomated software that can automatically calculate the ratio of the area of the MG’s dropout to the total MG area has been proposed [84]. Llorens–Quintana et al. [85] also developed a new algorithm for the automated detection of the MG area and objective analysis of the morphologic features of MGs. More recently, a fully automated algorithm that enables an objective, quantitative, and multiparametric assessment of meibography images using repeatable segmentation based on noise reduction and image contrast enhancement was introduced [86]. However, AI was not applied to these methods.

In 2012, Koh et al. [87] proposed computational methods for the automated detection of the width and length of MGs based on the infrared meibography images of 55 patients (26 ‘healthy’ and 29 ‘unhealthy’). A linear classifier was trained using features based on the widths and lengths of the MGs, which showed high accuracy (sensitivity, 97.9%, and specificity, 96.1%) [87]. Wang et al. [88] generated a DL-based algorithm using DNNs for the automated segmentation of the MG’s atrophy area and computation of the ratio of the atrophy area. Of note, this algorithm achieved a high accuracy of 95.6% for meiboscore grading, outperforming the clinical investigators by 16.0–40.6% [88]. It also attained a high 97.6% and 95.4% accuracy for the segmentations of the eyelid (97.6%) and atrophy (95.4%), respectively [88]. They also developed a DL-based approach for the automated segmentation of each MG region in meibography images and the analysis of their morphological features, which showed an 84.4% sensitivity and 71.7% specificity for the identification of ghost MGs [89]. In this algorithm, an SVM was applied for the analysis of the association between the morphological features of the MGs and ghost glands, which revealed that the low local contrast of the MG might be the primary indicator for ghost MGs [89]. Yeh et al. [90] introduced a DL-based network model using an unsupervised learning approach for the automated assessment of the severity of MG atrophy in meibography images, which demonstrated a high accuracy of 80.9% for the meiboscore grading, outperforming the clinical investigators by 25.9%. Setu et al. [91] proposed a DL-based method for MG segmentation by applying transfer learning with Inception-ResNet-v224, which is a pre-trained backbone. In this study, the baseline U-Net model was trained with transfer learning after pre-training using chest X-ray images to enhance transfer learning performance [91]. This algorithm achieved an AUC of 0.96, an average precision and recall of 83% and 81%, respectively, and an F-score of 84% [91]. A DL framework based on a Mask R-CNN was also developed for the segmentation of conjunctiva and MGs, which attained high accuracy in the segmentation of the conjunctiva (mean average precision [mAP] > 0.976, validation loss < 0.35) and MGs (mAP > 0.92, validation loss < 1.0) [92]. The evaluation of each image using this AI model took 480 ms, 21 times faster than that of ophthalmology specialists [92]. In 2022, Saha et al. [93] introduced a classification-based DL model that could enable the fast, automated, and objective assessment of the morphologic features of MGs, i.e., the segmentation of MGs, quantitative analysis of the area and ratio of MGs, and determination of the meiboscore. This AI model attained accuracies of 73.01% and 59.17% for the meiboscore classification on the validation set and on the images from independent centers, respectively, outperforming the accuracy of 53.44% by MGD specialists [93]. Moreover, this model removed the specular reflection from the original images using a generative adversarial network (GAN), which may allow for a distraction-free assessment by ophthalmologists [93]. These findings suggest that the integration of AI into the analysis of meibography images may enable the more precise and accurate monitoring of DED (Table 2). Although the AI-based analysis of meibography images enabled the quantitative analysis of MG changes, it cannot provide information regarding the changes in the three-dimensional structure of the MG. The development of devices capable of three-dimensional visualization of the MG is, therefore, needed.

### 3.4. Analysis of Interferometry Images Using AI

Tear film interferometry is a valuable tool for the diagnosis and monitoring of DED [82,94]. It allows visualization of the tear film layer and provides objective data on tear film properties, such as the thickness of the tear film and lipid layer, features of tear film break-up, and distribution and wetting patterns of the tear film with sequential blinking [82,94]. Previous studies have attempted to characterize the interference phenomena as a color texture pattern for automatic classification into interference pattern categories [95,96,97,98]. In these studies, various texture analysis methods and machine learning algorithms, including an SVM and multilayer perceptron, were used to analyze the interference patterns of the tear lipid layer for automatic classification [95,96,97,98].

Subsequently, da Cruz et al. [99] proposed an ML-based method for classifying tear interferometry images using texture analysis with phylogenetic diversity indices. The automated classification was tested using various algorithms, such as an RF, a multilayer perceptron, a naive Bayes, an SVM, a random tree, and a radial basis function network, with the RF classifier demonstrating the best results, with an accuracy of over 97%, an AUC of 0.99, an F-score of 0.97, and a Kappa index of 0.96 [99]. They also introduced a method for the automated classification of tear film interferometry images using feature extraction with phylogenetic diversity indices and Ripley’s K function [100]. Among the various ML algorithms, the best results were obtained with the RF classifier, with an accuracy of over 99%, an AUC of 0.999, an F-score of 0.996, and a Kappa index of 0.995 [100]. These findings suggest that an ML-based analysis of tear interferometry images can be a useful tool for screening DED. A small number of studies have been introduced so far, probably due to the technical difficulty in image processing and analysis. With further technological development, interferometry devices may provide images with an enhanced resolution and contrast, which may be more suitable for AI-based analysis.

### 3.5. Analysis of IVCM Images Using AI

IVCM is a non-invasive imaging modality that enables real-time visualization of the ocular surface tissue and changes in the ocular surface microstructures associated with DED [82,101]. However, although the IVCM image is objective, its interpretation may depend on the subjective assessment of the observers. The application of Al enables the objective and precise evaluation of ocular surface microstructures, such as the corneal nerves, epithelial cells, and dendritic cells, based on IVCM images [102,103,104,105].

In 2016, Al-Fahdawi et al. [102] introduced an Al system for the automated segmentation of the corneal nerve and the assessment of its morphological parameters, including nerve tortuosity, length, and thickness, using IVCM images, which is expected to be a powerful tool for the early detection of diabetic peripheral neuropathy. For a quantitative evaluation of diabetic polyneuropathy, Chen et al. [103] generated an automated algorithm based on a multilayer perceptron neural network and RF models for the detection and quantification of corneal nerve fibers using IVCM images. The automated quantification of the corneal nerves using this AI-based method showed a high correlation with the manually measured morphological features and similar results for the differential diagnosis of diabetic polyneuropathy, with repeatability and speed superior to manual quantification [103]. Williams et al. [104] recently introduced a DL algorithm employing a CNN with data augmentation for the automated quantification of the corneal sub-basal nerve plexus. This algorithm showed superior intraclass correlation coefficients for the corneal nerve parameters compared to a validated automated analysis software, ACCMetrics [104]. Moreover, it achieved a high AUC (0.83), specificity (0.87), and sensitivity (0.68) for the classification of participants with and without neuropathy, suggesting that AI-based algorithms can be a useful screening tool for peripheral neuropathy. Wei et al. [106] also developed an AI model for the automated segmentation and evaluation of sub-basal corneal nerve fibers in IVCM images using a CNN based on a DL algorithm. This model attained an AUC of 0.96 and mAP of 94%, with a substantially higher speed (32 images per second) than that of clinical investigators, suggesting that it can allow for the rapid and accurate assessment of changes in the corneal nerves in DED [106]. Subsequently, they analyzed the morphologic features of corneal sub-basal nerves in IVCM images using a DL model based on CNNs [107]. In this study, DED was associated with reduced density and the maximum length of corneal nerves measured using an AI algorithm [107]. The average corneal nerve density evaluated using a DL model had a negative correlation with corneal intrinsic aberrations, particularly higher-order aberrations [107]. Xu et al. [108] introduced deep transfer learning models that comprised pre-trained networks and an adaptation layer for automated detection of activated dendritic cells and inflammatory cells using IVCM images. In this study, the Inception-ResNet V2 transfer model achieved the best performance in identifying activated dendritic cells (AUC, 0.9646; accuracy, 0.9319; sensitivity, 0.8171; specificity, 0.9517; and G mean, 0.8872) and inflammatory cells (AUC, 0.9646; accuracy, 0.9767; sensitivity, 0.9174; specificity, 0.9931; and G mean, 0.9545), indicating that this AI model can be a powerful tool for the quantitative assessment of corneal inflammation in DED and monitoring of treatment responses [108].

A new DL algorithm based on GANs, an emerging DL model for the processing of medical images, was first introduced for the automated segmentation of corneal subbasal nerves in IVCM images in 2021 [109]. In comparison with the U-Net-based algorithm, the GAN-based algorithm showed a similar correlation and Bland–Altman analysis results. The GAN-based method demonstrated higher accuracy for the segmentation of corneal nerves in IVCM images, particularly in the applied images, compared to the U-Net-based method [109]. In 2022, a new DL-based algorithm that enabled the automated segmentation and evaluation of corneal nerve fibers (CNFs) and dendritic cells (DCs) separately in IVCM images based on U-Net and Mask R-CNN architectures, respectively, was produced by Setu et al. [110] In this study, both the CNF model (86.1% sensitivity and 90.1% specificity) and the DC model (89.37% precision, 94.43% recall, and 91.83% F1 score) showed reliable consistency with the manual evaluation and at a substantially higher speed, suggesting that the DL model has the potential to be integrated into the monitoring tools of DED using IVCM [110].

AI can also be helpful for the detection of MGD using IVCM [105,111]. Maruoka et al. [105] generated a DNN-based DL model for the detection of obstructive MGD using IVCM images. Their ensemble DL model achieved a high level of accuracy (AUC, 0.981; sensitivity, 92.1%; specificity, 98.8%), indicating that this model can enable the automated diagnosis of obstructive MGD based on IVCM images [105]. Zhang et al. [111] also introduced a DL model using a multilayer deep CNN for the differential diagnosis of MGD based on IVCM images. In this study, the DenseNet169 CNN model showed a high accuracy of over 97%, a sensitivity of 88.8%, and a specificity of 95.4%, indicating that the DL model has the potential to be a tool for the differential diagnosis of MGD [111].

The results of these studies suggest that AI-based analysis of IVCM images may provide information regarding changes in the corneal nerve, DCs, and MG. However, these microstructural changes may often have little correlation with the symptoms and signs of DED [62,63,64]. Therefore, a comprehensive approach comprising clinical data and information from various diagnostic modalities should be encouraged. Studies on the application of AI in the analysis of the IVCM images are summarized in Table 3.

### 3.6. Application of AI for Analysis of AS-OCT Images

AS-OCT can provide cross-sectional images of the tear meniscus and a quantitative assessment of tear film parameters, such as the height and area of the tear meniscus, which may be helpful for the diagnosis and monitoring of DED [112,113,114].

In 2020, Stegmann et al. [115] introduced a DL-based algorithm for the automatic segmentation of the lower tear meniscus using images taken with a custom-built AS-OCT system. In this study, 6658 images labeled by the thresholding-based segmentation algorithm were used to train deep CNNs with supervised learning [115]. The five-fold cross-validation showed a sensitivity of 96% and a specificity of 100%, indicating that DL-based segmentation of the tear meniscus can be a powerful tool for the evaluation of the tear film [115]. Using 158,220 images from 879 eyes of 478 participants, Elsawy et al. [116] developed a multi-disease DL diagnostic network based on AS-OCT images and revealed that it attained an AUC > 0.99, an area under precision-recall curve (AUPRC) > 0.96, and F1 scores > 0.90 for DED. AI can also be used to optimize the integration of data from AS-OCT images for screening and staging DED [117]. Edorh et al. [117] used RF regression to generate an optimal multivariable model for the diagnosis of DED that included data from wide corneal epithelial mapping using AS-OCT and validated the model using a bootstrapping method. This diagnostic algorithm showed high sensitivity (86.4%) and specificity (91.7%), suggesting that the integration of OCT corneal epithelial mapping data into a diagnostic algorithm using AI may improve the reliability of the diagnosis of DED [117]. Although a limited number of studies regarding the application of AI for the analysis of AS-OCT images have been conducted so far, the development of advanced devices, including high-resolution OCT, may enable the assessment of subtle changes in the ocular surface and tear film using AI [15].

## 4. Future Perspectives

The favorable results of the studies introduced in this review indicate that AI can be a critical tool for the management of DED in the future. AI can allow the diagnosis of DED and precise assessment of its severity by integrating the symptoms, signs, ocular and systemic risk factors, and results of various test devices. It can also enable precise monitoring of the treatment response of DED by detecting and analyzing subtle changes in dry eye signs, symptoms, and test results. With technological developments, images of anterior segment structures, meibomian glands, and the tear film can be obtained using mobile devices. By integrating and analyzing these images and clinical information provided by patients and primary healthcare providers, AI can enable telemedicine for the monitoring of DED and mass screening for the disease. AI can also improve the accuracy of the analysis of big data for the evaluation of the risk factors and prevalence of DED in the general population, which may enable the estimation of the risk of DED in each individual in the near future.

## 5. Conclusions

Advances in ML technology, particularly DL algorithms, have enabled the application of AI in various anterior segment diseases, including DED. AI is expected to be able to integrate the data from medical records, test results, and large population-based studies and provide optimized protocols for the diagnosis and management of DED [15]. The analysis of data obtained by imaging devices for DED, such as ASP, meibography, tear interferometry, IVCM, and AS-OCT, using AI may enable the precise and accurate assessment of the tests for DED, which would be critical for proper diagnosis and optimal treatment of the disease. The results of the studies introduced in this review are promising. With further development, integration of AI into the clinical approach for DED may be crucial for enhanced diagnostic and therapeutic performance.

## Figures and Tables

**Table 1:** List of abbreviations used in the review article.

Abbreviation	Name
AI	Artificial intelligence
ML	Machine learning
CML	Conventional machine learning
DL	Deep learning
SVM	Support vector machine
RF	Random forest
DT	Decision tree
ANN	Artificial neural network
RNN	Recurrent neural network
CNN	Convolutional neural network
OCT	Optical coherence tomography
ASP	Anterior segment photographs
AS-OCT	Anterior segment optical coherence tomography
IVCM	In vivo confocal microscopy
LASEK	Laser-assisted epithelial keratomileusis
LASIK	Laser in situ keratomileusis
SMILE	Small incision lenticular extraction
DMEK	Descemet membrane endothelial keratoplasty
AUC	Area under the receiver operating characteristic curve
DED	Dry eye disease
BUT	Break-up time
KNHANES	Korea National Health and Nutrition Examination Survey
LASSO	Least absolute shrinkage and selection operator
LR	logistic regression
RANSAC	RANdom SAmple Consensus
MRF	Material recovery facilities
MG	Meibomian gland
MGD	Meibomian gland dysfunction
mAP	Mean average precision
GAN	Generative adversarial network
CNF	Corneal nerve fiber
DC	Dendritic cell
AUPRC	Area under precision-recall curve

**Table 2:** Summary of the studies regarding application of AI for analysis of meibography images.

Study	Method (Protocol)	Number of Image Samples	Results
Koh et al. [87] (2012)	linear classifier	26 ‘healthy’ images 29 ‘unhealthy’ images	sensitivity, 97.9% specificity, 96.1%
Wang et al. [88] (2019)	deep neural network	497 for training and tuning 209 for evaluations	95.6% accuracy for meiboscore grading, 97.6% and 95.4% accuracy for eyelid and atrophy segmentations, respectively
Wang et al. [89] (2021)	DL model SVM	1039 for training and tuning 404 for evaluations	84.4% sensitivity and 71.7% specificity in identifying ghost meibomian gland
Yeh et al. [90] (2021)	unsupervised feature network learning	497 for network learning and tuning 209 for evaluations	80.9% accuracy for meiboscore grading, outperforming the clinical investigators by 25.9%
Setu et al. [91] (2021)	transfer learning with a pre-trained backbone training with U-net model no image pre-processing	502 and 126 for training and validation datasets, respectively 100 for comparison with manual annotations	The average precision score of 83% with AUC value of 0.96
Yu et al. [92] (2022)	Mask R-CNN	1878 for training and tuning 58 for evaluations	High accuracy in the identification of conjunctiva and meibomian glands (validation loss < 0.35 and <1.0, respectively, and mAP > 0.976 and >0.92, respectively) High speed that was 21 times faster than specialists
Saha et al. [93] (2022)	classification-based DL model generative adversarial network (GAN)	752 for training 189 for analyzing the performance 600 from an independent center for validation	73.01% and 59.17% accuracy for meiboscore classification on validation set and on images from independent center, respectively

**Table 3:** Summary of the studies regarding application of AI for analysis of IVCM images.

Study	Method (Protocol)	Number of Image Samples	Results
Al-Fahdawi et al. [102] (2016)	neural network RF SVM	498 for evaluation of segmentation 919 for evaluation of extracting morphometric features with clinical utility	rapid (13 s/image), robust and effective automated corneal nerve quantification
Chen et al. [103] (2017)	multi-layer perceptron neural network RF models	200 for training and validation 888 for testing	AUC of 0.77 and 72% sensitivity/specificity for identification of diabetic sensorimotor polyneuropathy
Williams et al. [104] (2020)	CNN with data augmentation	1698 for training 2137 for external validation	AUC of 0.83, specificity of 0.87, and sensitivity of 0.68 for detection of diabetic neuropathy
Wei et al. [106] (2020)	CNN	552 for training 139 for testing	High accuracy for corneal nerve segmentation (AUC 0.96) and a mean average precision (mAP) of 94%
Wei et al. [107] (2020)	CNN	229 eyes of 155 patients with DED 40 eyes of 20 healthy control	reduced density and maximum length of corneal nerve measured with AI algorithm had been association with DED
Xu et al. [108] (2021)	deep transfer learning network	3453 for training 558 for validation	AUC, 0.9646; sensitivity, 0.8171; specificity, 0.9517 in identifying activated dendritic cells AUC, 0.9901; sensitivity, 0.9174; specificity, 0.9931 in identifying inflammatory cells
Setu et al. [110] (2022)	U-Net for segmentation of corneal nerve fibers Mask R-CNN for segmentation of dendritic cells	U-Net (corneal nerve fibers) 1097 for training 122 for testing Dendritic cells 679 for training 75 for testing	Corneal nerve fibers model: 86.1% sensitivity and 90.1% specificity dendritic cell model: 89.37% precision, 94.43% recall, and 91.83% F1 score
Yildiz et al. [109] (2021)	U-net GANs	403 for training 102 for testing	The GAN-based algorithms showed higher accuracy than U-Net for automated corneal nerve segmentation based on IVCM images
Maruoka et al. [105] (2020)	Deep neural network	137 with obstructive MGD 84 with normal meibomian glands	High level of accuracy for detection of obstructive meibomian gland dysfunction (AUC 0.981, sensitivity 92.1%, and specificity 98.8%)
Zhang et al. [111] (2021)	multi-layer deep CNN	4985 for training 1663 for validation	excellent accuracy (over 97%) for differential diagnosis of MGD

## References

[1] Balyen L., Peto T. (2019). Promising Artificial Intelligence-Machine Learning-Deep Learning Algorithms in Ophthalmology. Asia Pac. J. Ophthalmol..

[2] Ting D.S.J., Foo V.H., Yang L.W.Y., Sia J.T., Ang M., Lin H., Chodosh J., Mehta J.S., Ting D.S.W. (2021). Artificial intelligence for anterior segment diseases: Emerging applications in ophthalmology. Br. J. Ophthalmol..

[3] LeCun Y., Bengio Y., Hinton G. (2015). Deep learning. Nature.

[4] Murdoch T.B., Detsky A.S. (2013). The inevitable application of big data to health care. JAMA.

[5] Ting D.S.W., Pasquale L.R., Peng L., Campbell J.P., Lee A.Y., Raman R., Tan G.S.W., Schmetterer L., Keane P.A., Wong T.Y. (2019). Artificial intelligence and deep learning in ophthalmology. Br. J. Ophthalmol..

[6] Storås A.M., Strümke I., Riegler M.A., Grauslund J., Hammer H.L., Yazidi A., Halvorsen P., Gundersen K.G., Utheim T.P., Jackson C.J. (2022). Artificial intelligence in dry eye disease. Ocul. Surf..

[7] Kapoor R., Walters S.P., Al-Aswad L.A. (2019). The current state of artificial intelligence in ophthalmology. Surv. Ophthalmol..

[8] Wu X., Liu L., Zhao L., Guo C., Li R., Wang T., Yang X., Xie P., Liu Y., Lin H. (2020). Application of artificial intelligence in anterior segment ophthalmic diseases: Diversity and standardization. Ann. Transl. Med..

[9] Ting D.S.W., Lee A.Y., Wong T.Y. (2019). An Ophthalmologist’s Guide to Deciphering Studies in Artificial Intelligence. Ophthalmology.

[10] De Fauw J., Ledsam J.R., Romera-Paredes B., Nikolov S., Tomasev N., Blackwell S., Askham H., Glorot X., O’Donoghue B., Visentin D. (2018). Clinically applicable deep learning for diagnosis and referral in retinal disease. Nat. Med..

[11] Ting D.S.W., Cheung C.Y., Lim G., Tan G.S.W., Quang N.D., Gan A., Hamzah H., Garcia-Franco R., San Yeo I.Y., Lee S.Y. (2017). Development and Validation of a Deep Learning System for Diabetic Retinopathy and Related Eye Diseases Using Retinal Images From Multiethnic Populations With Diabetes. JAMA.

[12] Taylor S., Brown J.M., Gupta K., Campbell J.P., Ostmo S., Chan R.V.P., Dy J., Erdogmus D., Ioannidis S., Kim S.J. (2019). Monitoring Disease Progression With a Quantitative Severity Scale for Retinopathy of Prematurity Using Deep Learning. JAMA Ophthalmol..

[13] Wagner S.K., Fu D.J., Faes L., Liu X.X., Huemer J., Khalid H., Ferraz D., Korot E., Kelly C., Balaskas K. (2020). Insights into Systemic Disease through Retinal Imaging-Based Oculomics. Transl. Vis. Sci. Technol..

[14] Schmidt-Erfurth U., Sadeghipour A., Gerendas B.S., Waldstein S.M., Bogunović H. (2018). Artificial intelligence in retina. Prog. Retin. Eye Res..

[15] Han S.B., Liu Y.C., Mohamed-Noriega K., Mehta J.S. (2021). Advances in Imaging Technology of Anterior Segment of the Eye. J. Ophthalmol..

[16] Mahesh Kumar S.V., Gunasundari R. (2018). Computer-Aided Diagnosis of Anterior Segment Eye Abnormalities using Visible Wavelength Image Analysis Based Machine Learning. J. Med. Syst..

[17] Liu X., Jiang J., Zhang K., Long E., Cui J., Zhu M., An Y., Zhang J., Liu Z., Lin Z. (2017). Localization and diagnosis framework for pediatric cataracts based on slit-lamp images using deep features of a convolutional neural network. PLoS ONE.

[18] Yang J.J., Li J., Shen R., He J., Bi J., Li Y., Zhang Q., Peng L., Wang Q. (2016). Exploiting ensemble learning for automatic cataract detection and grading. Comput. Methods Programs Biomed..

[19] Wu X., Huang Y., Liu Z., Lai W., Long E., Zhang K., Jiang J., Lin D., Chen K., Yu T. (2019). Universal artificial intelligence platform for collaborative management of cataracts. Br. J. Ophthalmol..

[20] Son K.Y., Ko J., Kim E., Lee S.Y., Kim M.J., Han J., Shin E., Chung T.Y., Lim D.H. (2022). Deep Learning-Based Cataract Detection and Grading from Slit-Lamp and Retro-Illumination Photographs: Model Development and Validation Study. Ophthalmol. Sci..

[21] Gutierrez L., Lim J.S., Foo L.L., Ng W.Y., Yip M., Lim G.Y.S., Wong M.H.Y., Fong A., Rosman M., Mehta J.S. (2022). Application of artificial intelligence in cataract management: Current and future directions. Eye Vis..

[22] Sramka M., Slovak M., Tuckova J., Stodulka P. (2019). Improving clinical refractive results of cataract surgery by machine learning. PeerJ.

[23] Li T., Stein J., Nallasamy N. (2022). AI-powered effective lens position prediction improves the accuracy of existing lens formulas. Br. J. Ophthalmol..

[24] Hung K.H., Lin C., Roan J., Kuo C.F., Hsiao C.H., Tan H.Y., Chen H.C., Ma D.H., Yeh L.K., Lee O.K. (2022). Application of a Deep Learning System in Pterygium Grading and Further Prediction of Recurrence with Slit Lamp Photographs. Diagnostics.

[25] Xu W., Jin L., Zhu P.Z., Yang W.H., Wu M.N. (2021). Implementation and Application of an Intelligent Pterygium Diagnosis System Based on Deep Learning. Front. Psychol..

[26] Fang X., Deshmukh M., Chee M.L., Soh Z.D., Teo Z.L., Thakur S., Goh J.H.L., Liu Y.C., Husain R., Mehta J. (2021). Deep learning algorithms for automatic detection of pterygium using anterior segment photographs from slit-lamp and hand-held cameras. Br. J. Ophthalmol..

[27] Kim J.H., Kim Y.J., Lee Y.J., Hyon J.Y., Han S.B., Kim K.G. (2022). Automated histopathological evaluation of pterygium using artificial intelligence. Br. J. Ophthalmol..

[28] Saini J.S., Jain A.K., Kumar S., Vikal S., Pankaj S., Singh S. (2003). Neural network approach to classify infective keratitis. Curr. Eye Res..

[29] Patel T.P., Prajna N.V., Farsiu S., Valikodath N.G., Niziol L.M., Dudeja L., Kim K.H., Woodward M.A. (2018). Novel Image-Based Analysis for Reduction of Clinician-Dependent Variability in Measurement of the Corneal Ulcer Size. Cornea.

[30] Liu Z., Shi Y., Zhan P., Zhang Y., Gong Y., Tang X. (2019). Automatic Corneal Ulcer Segmentation Combining Gaussian Mixture Modeling and Otsu Method. Conf. Proc. IEEE Eng. Med. Biol. Soc..

[31] Sun Q., Deng L., Liu J., Yuan J., Tang X. Patch-based deep convolutional neural network for corneal ulcer area segmentation. Proceedings of the Fetal, Infant and Ophthalmic Medical Image Analysis. International Workshop, FIFI 2017, and 4th International Workshop, OMIA 2017, Held in Conjunction with MICCAI 2017.

[32] Wu X., Tao Y., Qiu Q., Wu X. (2018). Application of image recognition-based automatic hyphae detection in fungal keratitis. Australas Phys. Eng. Sci. Med..

[33] Wu X., Qiu Q., Liu Z., Zhao Y., Zhang B., Zhang Y., Wu X., Ren J. (2018). Hyphae detection in fungal keratitis images with adaptive robust binary pattern. IEEE Access.

[34] Liu Z., Cao Y., Li Y., Xiao X., Qiu Q., Yang M., Zhao Y., Cui L. (2019). Automatic diagnosis of fungal keratitis using data augmentation and image fusion with deep convolutional neural network. Comput. Methods Programs Biomed..

[35] Ouabida E., Essadike A., Bouzid A. (2018). Automated segmentation of ophthalmological images by an optical based approach for early detection of eye tumor growing. Phys. Med..

[36] Ambrósio R., Lopes B.T., Faria-Correia F., Salomão M.Q., Bühren J., Roberts C.J., Elsheikh A., Vinciguerra R., Vinciguerra P. (2017). Integration of Scheimpflug-Based Corneal Tomography and Biomechanical Assessments for Enhancing Ectasia Detection. J. Refract. Surg..

[37] Lopes B.T., Ramos I.C., Salomão M.Q., Guerra F.P., Schallhorn S.C., Schallhorn J.M., Vinciguerra R., Vinciguerra P., Price F.W., Price M.O. (2018). Enhanced Tomographic Assessment to Detect Corneal Ectasia Based on Artificial Intelligence. Am. J. Ophthalmol..

[38] Ferreira-Mendes J., Lopes B.T., Faria-Correia F., Salomao M.Q., Rodrigues-Barros S., Ambrosio R. (2019). Enhanced Ectasia Detection Using Corneal Tomography and Biomechanics. Am. J. Ophthalmol..

[39] Yousefi S., Yousefi E., Takahashi H., Hayashi T., Tampo H., Inoda S., Arai Y., Asbell P. (2018). Keratoconus severity identification using unsupervised machine learning. PLoS ONE.

[40] Lavric A., Valentin P. (2019). KeratoDetect: Keratoconus Detection Algorithm Using Convolutional Neural Networks. Comput. Intell. Neurosci..

[41] Accardo P.A., Pensiero S. (2002). Neural network-based system for early keratoconus detection from corneal topography. J. Biomed. Inform..

[42] Kamiya K., Ayatsuka Y., Kato Y., Fujimura F., Takahashi M., Shoji N., Mori Y., Miyata K. (2019). Keratoconus detection using deep learning of colour-coded maps with anterior segment optical coherence tomography: A diagnostic accuracy study. BMJ Open.

[43] Valdes-Mas M.A., Martin-Guerrero J.D., Ruperez M.J., Pastor F., Dualde C., Monserrat C., Peris-Martinez C. (2014). A new approach based on Machine Learning for predicting corneal curvature (K1) and astigmatism in patients with keratoconus after intracorneal ring implantation. Comput. Methods Programs Biomed..

[44] Saad A., Gatinel D. (2016). Combining Placido and Corneal Wavefront Data for the Detection of Forme Fruste Keratoconus. J. Refract Surg..

[45] Yoo T.K., Ryu I.H., Lee G., Kim M.J., Tchah H. (2015). Adopting machine learning to automatically identify candidate patients for corneal refractive surgery. J. Refract Surg..

[46] Cui T., Wang Y., Ji S., Li Y., Hao W., Zou H., Jhanji V. (2020). Applying Machine Learning Techniques in Nomogram Prediction and Analysis for SMILE Treatment. Am. J. Ophthalmol..

[47] Joseph N., Kolluru C., Benetz B.A.M., Menegay H.J., Lass J.H., Wilson D.L. (2020). Quantitative and qualitative evaluation of deep learning automatic segmentations of corneal endothelial cell images of reduced image quality obtained following cornea transplant. J. Med. Imaging.

[48] Kolluru C., Benetz B.A., Joseph N., Menegay H.J., Lass J.H., Wilson D. (2019). Machine learning for segmenting cells in corneal endothelium images. Proc. SPIE Int. Soc. Opt. Eng..

[49] Vigueras-Guillen J.P., Andrinopoulou E.R., Engel A., Lemij H.G., van Rooij J., Vermeer K.A., van Vliet L.J. (2018). Corneal Endothelial Cell Segmentation by Classifier-Driven Merging of Oversegmented Images. IEEE Trans. Med. Imaging.

[50] Vigueras-Guillen J.P., van Rooij J., Lemij H.G., Vermeer K.A., van Vliet L.J. Convolutional neural network-based regression for biomarker estimation in corneal endothelium microscopy images. Proceedings of the 2019 41st Annual International Conference of the IEEE Engineering in Medicine and Biology Society (EMBC).

[51] Heinzelmann S., Daniel M.C., Maier P.C., Reinhard T., Böhringer D. (2019). Automated Cell Counting Using “Deep Learning” in Donor Corneas from Organ Culture Achieves High Precision and Accuracy. Klin. Monbl. Augenheilkd..

[52] Vigueras-Guillén J.P., Sari B., Goes S.F., Lemij H.G., van Rooij J., Vermeer K.A., van Vliet L.J. (2019). Fully convolutional architecture vs. sliding-window CNN for corneal endothelium cell segmentation. BMC Biomed. Eng..

[53] Treder M., Lauermann J.L., Alnawaiseh M., Eter N. (2019). Using Deep Learning in Automated Detection of Graft Detachment in Descemet Membrane Endothelial Keratoplasty: A Pilot Study. Cornea.

[54] Hayashi T., Tabuchi H., Masumoto H., Morita S., Oyakawa I., Inoda S., Kato N., Takahashi H. (2020). A Deep Learning Approach in Rebubbling After Descemet’s Membrane Endothelial Keratoplasty. Eye Contact Lens..

[55] Han S.B., Yang H.K., Hyon J.Y., Wee W.R. (2017). Association of dry eye disease with psychiatric or neurological disorders in elderly patients. Clin. Interv. Aging.

[56] Stapleton F., Alves M., Bunya V.Y., Jalbert I., Lekhanont K., Malet F., Na K.S., Schaumberg D., Uchino M., Vehof J. (2017). TFOS DEWS II Epidemiology Report. Ocul. Surf..

[57] Yamanishi R., Uchino M., Uchino Y., Kawashima M., Dogru M., Yokoi N., Tsubota K. (2020). Changes in Distribution of Dry Eye Diagnostic Status Among Visual Display Terminal Workers According to the Revised Criteria of the Asia Dry Eye Society. Cornea.

[58] Craig J.P., Nichols K.K., Akpek E.K., Caffery B., Dua H.S., Joo C.K., Liu Z., Nelson J.D., Nichols J.J., Tsubota K. (2017). TFOS DEWS II Definition and Classification Report. Ocul. Surf..

[59] Miljanovic B., Trivedi K.A., Dana M.R., Gilbard J.P., Buring J.E., Schaumberg D.A. (2005). Relation between dietary n-3 and n-6 fatty acids and clinically diagnosed dry eye syndrome in women. Am. J. Clin. Nutr..

[60] Uchino M., Schaumberg D.A., Dogru M., Uchino Y., Fukagawa K., Shimmura S., Satoh T., Takebayashi T., Tsubota K. (2008). Prevalence of dry eye disease among Japanese visual display terminal users. Ophthalmology.

[61] Sayegh R.R., Yu Y., Farrar J.T., Kuklinski E.J., Shtein R.M., Asbell P.A., Maguire M.G. (2021). Ocular Discomfort and Quality of Life Among Patients in the Dry Eye Assessment and Management Study. Cornea.

[62] Nichols K.K., Nichols J.J., Mitchell G.L. (2004). The lack of association between signs and symptoms in patients with dry eye disease. Cornea.

[63] Vehof J., Sillevis Smitt-Kamminga N., Nibourg S.A., Hammond C.J. (2017). Predictors of Discordance between Symptoms and Signs in Dry Eye Disease. Ophthalmology.

[64] Ong E.S., Felix E.R., Levitt R.C., Feuer W.J., Sarantopoulos C.D., Galor A. (2018). Epidemiology of discordance between symptoms and signs of dry eye. Br. J. Ophthalmol..

[65] Han S.B., Hyon J.Y., Woo S.J., Lee J.J., Kim T.H., Kim K.W. (2011). Prevalence of dry eye disease in an elderly Korean population. Arch. Ophthalmol..

[66] Nichols K.K., Mitchell G.L., Zadnik K. (2004). The repeatability of clinical measurements of dry eye. Cornea.

[67] Savini G., Prabhawasat P., Kojima T., Grueterich M., Espana E., Goto E. (2008). The challenge of dry eye diagnosis. Clin. Ophthalmol..

[68] Grus F.H., Augustin A.J. (1999). Analysis of tear protein patterns by a neural network as a diagnostical tool for the detection of dry eyes. Electrophoresis.

[69] Nam S.M., Peterson T.A., Butte A.J., Seo K.Y., Han H.W. (2020). Explanatory Model of Dry Eye Disease Using Health and Nutrition Examinations: Machine Learning and Network-Based Factor Analysis From a National Survey. JMIR Med. Inform..

[70] Dros J.T., Bos I., Bennis F.C., Wiegersma S., Paget J., Seghieri C., Barrio Cortés J., Verheij R.A. (2022). Detection of primary Sjögren’s syndrome in primary care: Developing a classification model with the use of routine healthcare data and machine learning. BMC Prim. Care.

[71] Yedidya T., Hartley R., Guillon J.-P., Kanagasingam Y., Ayache N., Ourselin S., Maeder A. (2007). Automatic Dry Eye Detection. Medical Image Computing and Computer-Assisted Intervention—MICCAI 2007.

[72] Yedidya T., Carr P., Hartley R., Guillon J.P. (2009). Enforcing monotonic temporal evolution in dry eye images. Med. Image Comput. Comput. Assist. Interv..

[73] Zheng Q., Wang L., Wen H., Ren Y., Huang S., Bai F., Li N., Craig J.P., Tong L., Chen W. (2022). Impact of Incomplete Blinking Analyzed Using a Deep Learning Model With the Keratograph 5M in Dry Eye Disease. Transl. Vis. Sci. Technol..

[74] Wang Y., Jia X., Wei S., Li X. (2022). A deep learning model established for evaluating lid margin signs with colour anterior segment photography. Eye.

[75] Chun Y.S., Yoon W.B., Kim K.G., Park I.K. (2014). Objective assessment of corneal staining using digital image analysis. Investig. Ophthalmol. Vis. Sci..

[76] Pellegrini M., Bernabei F., Moscardelli F., Vagge A., Scotto R., Bovone C., Scorcia V., Giannaccare G. (2019). Assessment of Corneal Fluorescein Staining in Different Dry Eye Subtypes Using Digital Image Analysis. Transl. Vis. Sci. Technol..

[77] Park I.K., Chun Y.S., Kim K.G., Yang H.K., Hwang J.M. (2013). New clinical grading scales and objective measurement for conjunctival injection. Investig. Ophthalmol. Vis. Sci..

[78] Han S.B., Jeon H.S., Kim M., Lee S.J., Yang H.K., Hwang J.M., Kim K.G., Hyon J.Y., Wee W.R. (2016). Risk Factors for Recurrence After Pterygium Surgery: An Image Analysis Study. Cornea.

[79] Han S.B., Jeon H.S., Kim M., Lee S.J., Yang H.K., Hwang J.M., Kim K.G., Hyon J.Y., Wee W.R. (2016). Quantification of Astigmatism Induced by Pterygium Using Automated Image Analysis. Cornea.

[80] Yang H.K., Lee Y.J., Hyon J.Y., Kim K.G., Han S.B. (2020). Efficacy of bevacizumab injection after pterygium excision and limbal conjunctival autograft with limbal fixation suture. Graefes Arch. Clin. Exp. Ophthalmol..

[81] Kim Y.J., Yang H.K., Lee Y.J., Hyon J.Y., Kim K.G., Han S.B. (2020). Efficacy of a new automated method for quantification of corneal neovascularisation. Br. J. Ophthalmol..

[82] Han S.B., Liu Y.C., Mohamed-Noriega K., Tong L., Mehta J.S. (2020). Objective Imaging Diagnostics for Dry Eye Disease. J. Ophthalmol..

[83] Ban Y., Shimazaki-Den S., Tsubota K., Shimazaki J. (2013). Morphological evaluation of meibomian glands using noncontact infrared meibography. Ocul. Surf..

[84] Binotti W.W., Bayraktutar B., Ozmen M.C., Cox S.M., Hamrah P. (2020). A Review of Imaging Biomarkers of the Ocular Surface. Eye Contact Lens.

[85] Llorens-Quintana C., Rico-Del-Viejo L., Syga P., Madrid-Costa D., Iskander D.R. (2019). A Novel Automated Approach for Infrared-Based Assessment of Meibomian Gland Morphology. Transl. Vis. Sci. Technol..

[86] Xiao P., Luo Z., Deng Y., Wang G., Yuan J. (2021). An automated and multiparametric algorithm for objective analysis of meibography images. Quant Imaging Med. Surg..

[87] Koh Y.W., Celik T., Lee H.K., Petznick A., Tong L. (2012). Detection of meibomian glands and classification of meibography images. J. Biomed. Opt..

[88] Wang J., Yeh T.N., Chakraborty R., Yu S.X., Lin M.C. (2019). A Deep Learning Approach for Meibomian Gland Atrophy Evaluation in Meibography Images. Transl. Vis. Sci. Technol..

[89] Wang J., Li S., Yeh T.N., Chakraborty R., Graham A.D., Yu S.X., Lin M.C. (2021). Quantifying Meibomian Gland Morphology Using Artificial Intelligence. Optom. Vis. Sci..

[90] Yeh C.H., Yu S.X., Lin M.C. (2021). Meibography Phenotyping and Classification From Unsupervised Discriminative Feature Learning. Transl. Vis. Sci. Technol..

[91] Setu M.A.K., Horstmann J., Schmidt S., Stern M.E., Steven P. (2021). Deep learning-based automatic meibomian gland segmentation and morphology assessment in infrared meibography. Sci. Rep..

[92] Yu Y., Zhou Y., Tian M., Zhou Y., Tan Y., Wu L., Zheng H., Yang Y. (2022). Automatic identification of meibomian gland dysfunction with meibography images using deep learning. Int. Ophthalmol..

[93] Saha R.K., Chowdhury A.M.M., Na K.S., Hwang G.D., Eom Y., Kim J., Jeon H.G., Hwang H.S., Chung E. (2022). Automated quantification of meibomian gland dropout in infrared meibography using deep learning. Ocul. Surf..

[94] Arita R., Morishige N., Fujii T., Fukuoka S., Chung J.L., Seo K.Y., Itoh K. (2016). Tear Interferometric Patterns Reflect Clinical Tear Dynamics in Dry Eye Patients. Investig. Ophthalmol. Vis. Sci..

[95] Remeseiro B., Penas M., Mosquera A., Novo J., Penedo M.G., Yebra-Pimentel E. (2012). Statistical comparison of classifiers applied to the interferential tear film lipid layer automatic classification. Comput. Math Methods Med..

[96] Remeseiro B., Penas M., Barreira N., Mosquera A., Novo J., García-Resúa C. (2013). Automatic classification of the interferential tear film lipid layer using colour texture analysis. Comput. Methods Programs Biomed..

[97] Remeseiro B., Bolon-Canedo V., Peteiro-Barral D., Alonso-Betanzos A., Guijarro-Berdiñas B., Mosquera A., Penedo M.G., Sánchez-Maroño N. (2014). A methodology for improving tear film lipid layer classification. IEEE J. Biomed. Health Inform..

[98] Peteiro-Barral D., Remeseiro B., Méndez R., Penedo M.G. (2017). Evaluation of an automatic dry eye test using MCDM methods and rank correlation. Med. Biol. Eng. Comput..

[99] da Cruz L.B., Souza J.C., de Sousa J.A., Santos A.M., de Paiva A.C., de Almeida J.D.S., Silva A.C., Junior G.B., Gattass M. (2020). Interferometer eye image classification for dry eye categorization using phylogenetic diversity indexes for texture analysis. Comput. Methods Programs Biomed..

[100] da Cruz L.B., Souza J.C., de Paiva A.C., de Almeida J.D.S., Junior G.B., Aires K.R.T., Silva A.C., Gattass M. (2020). Tear Film Classification in Interferometry Eye Images Using Phylogenetic Diversity Indexes and Ripley’s K Function. IEEE J. Biomed. Health Inform..

[101] Villani E., Bonsignore F., Cantalamessa E., Serafino M., Nucci P. (2020). Imaging Biomarkers for Dry Eye Disease. Eye Contact. Lens.

[102] Al-Fahdawi S., Qahwaji R., Al-Waisy A.S., Ipson S., Malik R.A., Brahma A., Chen X. (2016). A fully automatic nerve segmentation and morphometric parameter quantification system for early diagnosis of diabetic neuropathy in corneal images. Comput. Methods Programs Biomed..

[103] Chen X., Graham J., Dabbah M.A., Petropoulos I.N., Tavakoli M., Malik R.A. (2017). An Automatic Tool for Quantification of Nerve Fibers in Corneal Confocal Microscopy Images. IEEE Trans. Biomed. Eng..

[104] Williams B.M., Borroni D., Liu R., Zhao Y., Zhang J., Lim J., Ma B., Romano V., Qi H., Ferdousim M. (2020). An artificial intelligence-based deep learning algorithm for the diagnosis of diabetic neuropathy using corneal confocal microscopy: A development and validation study. Diabetologia.

[105] Maruoka S., Tabuchi H., Nagasato D., Masumoto H., Chikama T., Kawai A., Oishi N., Maruyama T., Kato Y., Hayashi T. (2020). Deep Neural Network-Based Method for Detecting Obstructive Meibomian Gland Dysfunction With in Vivo Laser Confocal Microscopy. Cornea.

[106] Wei S., Shi F., Wang Y., Chou Y., Li X. (2020). A Deep Learning Model for Automated Sub-Basal Corneal Nerve Segmentation and Evaluation Using In Vivo Confocal Microscopy. Transl. Vis. Sci. Technol..

[107] Jing D., Liu Y., Chou Y., Jiang X., Ren X., Yang L., Su J., Li X. (2022). Change patterns in the corneal sub-basal nerve and corneal aberrations in patients with dry eye disease: An artificial intelligence analysis. Exp Eye Res.

[108] Xu F., Qin Y., He W., Huang G., Lv J., Xie X., Diao C., Tang F., Jiang L., Lan R. (2021). A deep transfer learning framework for the automated assessment of corneal inflammation on in vivo confocal microscopy images. PLoS ONE.

[109] Yildiz E., Arslan A.T., Yildiz Tas A., Acer A.F., Demir S., Sahin A., Erol Barkana D. (2021). Generative Adversarial Network Based Automatic Segmentation of Corneal Subbasal Nerves on In Vivo Confocal Microscopy Images. Transl. Vis. Sci. Technol..

[110] Setu M.A.K., Schmidt S., Musial G., Stern M.E., Steven P. (2022). Segmentation and Evaluation of Corneal Nerves and Dendritic Cells From In Vivo Confocal Microscopy Images Using Deep Learning. Transl. Vis. Sci. Technol..

[111] Zhang Y.Y., Zhao H., Lin J.Y., Wu S.N., Liu X.W., Zhang H.D., Shao Y., Yang W.F. (2021). Artificial Intelligence to Detect Meibomian Gland Dysfunction From in-vivo Laser Confocal Microscopy. Front. Med..

[112] Chan T.C.Y., Wan K.H., Shih K.C., Jhanji V. (2018). Advances in dry eye imaging: The present and beyond. Br. J. Ophthalmol..

[113] Ramos J.L., Li Y., Huang D. (2009). Clinical and research applications of anterior segment optical coherence tomography—A review. Clin. Exp. Ophthalmol..

[114] Han S.B., Liu Y.C., Noriega K.M., Mehta J.S. (2016). Applications of Anterior Segment Optical Coherence Tomography in Cornea and Ocular Surface Diseases. J. Ophthalmol..

[115] Stegmann H., Werkmeister R.M., Pfister M., Garhöfer G., Schmetterer L., Dos Santos V.A. (2020). Deep learning segmentation for optical coherence tomography measurements of the lower tear meniscus. Biomed. Opt. Express..

[116] Elsawy A., Eleiwa T., Chase C., Ozcan E., Tolba M., Feuer W., Abdel-Mottaleb M., Abou Shousha M. (2021). Multidisease Deep Learning Neural Network for the Diagnosis of Corneal Diseases. Am. J. Ophthalmol..

[117] Edorh N.A., El Maftouhi A., Djerada Z., Arndt C., Denoyer A. (2022). New model to better diagnose dry eye disease integrating OCT corneal epithelial mapping. Br. J. Ophthalmol..

